# Vermicompost Supply Enhances Fragrant-Rice Yield by Improving Soil Fertility and Eukaryotic Microbial Community Composition under Environmental Stress Conditions

**DOI:** 10.3390/microorganisms12061252

**Published:** 2024-06-20

**Authors:** Anas Iqbal, Quaid Hussain, Zhaowen Mo, Tian Hua, Abd El-Zaher M. A. Mustafa, Xiangru Tang

**Affiliations:** 1 State Key Laboratory for Conservation and Utilization of Subtropical Agro-Bioresources, College of Agriculture, South China Agricultural University, Guangzhou 510642, China; anasiqbal@scau.edu.cn (A.I.);; 2 Guangzhou Key Laboratory for Science and Technology of Fragrant Rice, Guangzhou 510642, China; 3 College of Life Science and Oceanography, Shenzhen University, Shenzhen 518060, China; quaid_hussain@yahoo.com; 4 Department of Botany and Microbiology, College of Science, King Saud University, Riyadh P.O. Box 11451, Saudi Arabia; amus@ksu.edu.sa

**Keywords:** fragrant rice, heavy metals, microbial biomass, paddy field, soil environmental factors, soil microbial community, vermicompost

## Abstract

Heavy-metal contamination in agricultural soil, particularly of cadmium (Cd), poses serious threats to soil biodiversity, rice production, and food safety. Soil microbes improve soil fertility by regulating soil organic matter production, plant nutrient accumulation, and pollutant transformation. Addressing the impact of Cd toxicity on soil fungal community composition, soil health, and rice yield is urgently required for sustainable rice production. Vermicompost (VC) is an organic fertilizer that alleviates the toxic effects of Cd on soil microbial biodiversity and functionality and improves crop productivity sustainably. In the present study, we examined the effects of different doses of VC (i.e., 0, 3, and 6 tons ha^−1^) and levels of Cd stress (i.e., 0 and 25 mg Cd kg^−1^) on soil biochemical attributes, soil fungal community composition, and fragrant-rice grain yield. The results showed that the Cd toxicity significantly reduced soil fertility, eukaryotic microbial community composition and rice grain yield. However, the VC addition alleviated the Cd toxicity and significantly improved the soil fungal community; additionally, it enhanced the relative abundance of *Ascomycota*, *Chlorophyta*, *Ciliophora*, *Basidiomycota*, and *Glomeromycta* in Cd-contaminated soils. Moreover, the VC addition enhanced the soil’s chemical attributes, including soil pH, soil organic carbon (SOC), available nitrogen (AN), total nitrogen (TN), and microbial biomass C and N, compared to non-VC treated soil under Cd toxicity conditions. Similarly, the VC application significantly increased rice grain yield and decreased the Cd uptake in rice. One possible explanation for the reduced Cd uptake in plants is that VC amendments influence the soil’s biological properties, which ultimately reduces soil Cd bioavailability and subsequently influences the Cd uptake and accumulation in rice plants. RDA analysis determined that the leading fungal species were highly related to soil environmental attributes and microbial biomass C and N production. However, the relative abundance levels of *Ascomycota*, *Basidiomycota*, and *Glomeromycta* were strongly associated with soil environmental variables. Thus, the outcomes of this study reveal that the use of VC in Cd-contaminated soils could be useful for sustainable rice production and safe utilization of Cd-polluted soil.

## 1. Introduction

All heavy metals, especially Cd, a highly toxic metal, cause significant damage and may present enhanced risks to human health [1,2]. Human activities, i.e., mining and smelting, agricultural practices, and the application of pesticides and insecticides, have impacted the high ratio of heavy metals in modern agricultural techniques [3,4,5]. Cd is typically non-recyclable and challenging to eliminate from the soil, and it can be moved to food grains through the soil–plant–food cycle, negatively impacting human health [5,6,7]. Researchers took great interest in Cd pollution in arable soil due to its high mobility, toxicity, and long life in living organisms [5]. A meta-analysis study shows that 29.5% of arable soils in China are polluted with Cd [8]. Moreover, Cd inputs into the soil also affect soil biodiversity and the functioning of its allied ecosystems [9].

Soil microbes are vital for maintaining soil health and decomposing organic matter and metal contaminants [10]. Microbes form the soil environment, which then participates in soil’s biological activities and serves as a link for the exchange of materials between soil and plants [11,12]. They are sensitive to soil-related alterations, which have substantial impacts on the functions of the ecosystem [13]. In addition, heavy-metal pollution can have adverse effects on the development, survival, diversity, and composition of soil microbes [14]. Cd toxicity was identified as a key element influencing changes in soil fungus community assembly [15]. According to Song et al. [16], Cd stress reduced the microbial community richness in underlying soils. Thus, it is crucial to repair Cd-contaminated agricultural soils. Moreover, soil enzymes are susceptible to heavy-metal stress and are linked to soil processes including the C, N, and P cycles [17]. The activity of enzymes is therefore used as a bioindicator for soil and ecosystem wellness [13,18]. Furthermore, soil health and heavy-metal type influence enzyme activity responses to heavy-metal stress [19]. Soil pH significantly affects β-glucosidase and acid phosphatase enzyme activity [20,21]. Although the effects of soil parameters on enzyme activity have been extensively explored, the cause-and-effect relationship between soil variables and fungal community under Cd toxicity remains unexplained.

The influence of organic fertilizer on soil’s chemical and biological properties has recently become an interesting subject. VC is the product of the breakdown of organic materials through the composting process; it is commonly used as a soil amendment or organic fertilizer to enhance the soil’s health and crop yields due to its high humified content of organic matter and excellent abilities in ventilation, water retention, porosity, drainage, and microbial reproduction, making it an ideal soil conditioner [22,23]. In addition, VC has a high particle surface area, resulting in multiple microsites for soil fungal activity and solid nutrient preservation [19]. Therefore, the majority of plant macro- and micronutrients are readily available [24]. Furthermore, research has shown that applying organic materials to soil enhances biomass C and N and the relevant metabolic ratios, all of which represent soil-microbe activities [17,18]. Enhanced microbial populations and activity after the addition of organic matter to arable soils have been noticed [19]. In this regard, VC, which stabilizes organic materials through the interaction of microbes and earthworms, has been considered a helpful asset for soil restoration and the improvement of C amounts and soil health [25]. Furthermore, Zhen et al. [25] stated that using VC in agricultural land increased soil productivity, crop production, and soil microbial diversity. However, there is a paucity of understanding of how different dosages of VC affect soil fertility, soil microbial community composition, and diversity in Cd-contaminated soil.

Additionally, in situ, stabilization technology procedures have shown that VC is an environmentally friendly means for increasing soil health and crop production, while also immobilizing Cd [26,27]. VC, a nutrient-rich organic natural fertilizer, has received increasing attention for countering Cd content in heavy-metal-contaminated soil [28,29]. VC is more effective than plant compost in lowering Cd level in soil and its uptake in plants due to its maximum capacity, high specific area, strong cation exchangeability, and enrichment in the active structural group [30,31,32]. Moreover, VC application improves the germination, growth, and grain yield of high-value crops [33]. VC comprises plant-growth regulation materials, i.e., humic acid, auxins, gibberellins, and cytokinins [34,35], which can account for enhanced cereal crop production and yield [23]. These crop growth-regulating materials are formed by the action of soil microorganisms like bacteria, fungi, and actinomycetes, as well as earthworms [35]. Hence, VC reveals crop growth and production effects comparable to those displayed by soil-applied chemical fertilizers, hormones, or crop growth regulators.

Rice is a staple food around the globe and is a primary source of food implicated in soil Cd intake [36,37]. Cd potentially polluted farming land in China, and its toxicity poses a significant threat to rice grain quality and nutritional value, adversely impacting human health and crop production [38,39]. Cd is commonly found in soil and is subject to uptake by rice plants, subsequently accumulating in the grains [40]. High levels of Cd in rice grains not only diminish their quality by altering taste and texture but also reduce their nutritional value [41]. The Cd-contaminated rice grain can lead to health issues such as kidney damage, skeletal abnormalities, and even certain types of cancer [42]. In addition, Cd competes with essential nutrients such as zinc and iron, decreasing their bioavailability in rice grain, thus exacerbating micronutrient deficiencies in populations heavily reliant on rice as a staple food [43]. Therefore, addressing Cd toxicity in rice cultivation is crucial for ensuring the food safety and nutritional security of communities globally. In this study, we applied VC as a composite material in Cd-contaminated soil to counteract the negative impacts of Cd on soil fertility, soil microbial biodiversity, and rice production. To the best of our knowledge, there is limited information regarding the measured variables in this study in the context of soil and fragrant rice crops relative to different VC amendments under Cd toxicity conditions. The main objectives of the study were: (1) to investigate the impacts of VC on soil environmental parameters and soil microbial biomass C and N; (2) to assess the role of VC in improving soil microbial community structure and composition and its relationship with soil biochemical parameters; and (3) to explore the effect of VC application on fragrant-rice yield and understand the safe production of fragrant rice under Cd toxicity. The current study hypothesized that applying VC would improve soil quality and microbial community richness and composition in the presence of Cd toxicity. This work aimed to produce a conceptual framework for safe and sustainable crop production in Cd-contaminated soils.

## 2. Materials and Methods

### 2.1. Research Location

The study was carried out at the Research Station of South China Agriculture University, Guangzhou, China. The experimental soil (0–20 cm) was slightly acidic, consisting of 11.5 g kg^−1^ SOC, 89.65 mg kg^−1^ AN, 1.22 g kg^−1^ TN, and 0.96 g kg^−1^ total phosphorous (TP); the detailed soil physiochemical composition is shown in the Supplementary Material, in Table S1. This site has a humid subtropical monsoon climatic condition; the details of the weather conditions are shown in Table S2.

### 2.2. Experimental Details

A pot study was conducted in the late rice growing season (July–November) of 2022, featuring a complete block design with six treatments and three replications (Figure S1). The soil was collected to a depth of 20 cm from a clean paddy field, dried, powdered, and put in plastic pots. Further, it was ensured that all pots contained the same size and weight of soil to minimize experimental error. The applied VC was manufactured by Hubei Tianhenjia Biological Environmental Protection Technology Co., Ltd., Wuxue City, Hubei Province, China; it consisted of 34.90% organic matter, 1.48% TN, 2.76% P_2_O_2_, and 1.00% K_2_O, and had a pH of 7.6. It was used at different doses, i.e., V1 = 0 t ha^−1^, V2 = 3 t ha^−1^, and V3 = 6 t ha^−1^ as a basal dose, based on our research group’s previous study [44], which had optimized the fertilizer rate of VC. Two concentrations of Cd (i.e., Cd1, 0; and Cd2 25 mg Cd kg^−1^ soil using CdCl_2_·2.5 H_2_O) were used. The detailed treatments are shown in Table 1. Ten days before the seedling transplantation, the Cd and VC were thoroughly mixed and put in pots. The seeds of the fragrant-rice cultivar Meixiangzhan-2 were used as a test crop and cultivated in a plastic pot, with each pot containing three hills. The seedlings were transplanted into pots in mid-July, and the rice crops were harvested in mid-November. Uniform flooding irrigation was maintained from the planting of seedlings to physiological maturity to establish anaerobic conditions in the pots. Usual farming practices, such as insecticide and pesticide application, were applied in all treatments.

### 2.3. Sampling and Analysis

#### 2.3.1. Chemical Properties of Soil and Microbial Biomass C and N

A core sampler was used to collect soil samples to a depth of 20 cm from each pot before seedling emergence and after harvest. We collected three replicated soil samples for each measuring variable, and then the samples were further divided into two parts, half for nutrient assessments and the other for molecular analysis, both stored at −80 °C. Wang et al. [45] described the utilized K_2_Cr_2_O_7_-H_2_SO_4_ oxidation method for determining soil organic carbon (SOC). Furthermore, the Ohyama et al. [46] approach was employed for TN. The total N was estimated with Jackson’s [47] micro-Kjeldahl method. Finally, the soil’s AN, TP, TK, and pH were determined using the Lu [48] methods. The fumigation-extract approach was used to measure microbial biomass N (MBN), as described by Brookes et al. [49], and microbial biomass C (MBC), as described by Vance et al. [50].

#### 2.3.2. Soil DNA Extraction and PCR Amplification

The FastDNA Spin Kit for Soil (MP Biomedicals, LLC., Solon, OH, USA) was used to extract DNA from 0.5 g of fresh soil, following the manufacturer’s instructions. Electrophoresis and a NanoDrop^®^ND 2000 UV vis spectrophotometer (NanoDrop Technologies, Wilmington, DE, USA) were used to assess the DNA integrity and concentrations; samples were then stored at −20 °C. White et al. [51] have described the use of primers ITS 5 F (5′-GGAAGTAAAAGTCGTAACAAGG-3′) and ITS 2 R (5′-GCTGCGTTCTTCATCGATGC-3′) to amplify the ITS1 region. PCR mixtures (10 µL) comprised 20 µL TransStart Top Green qPCR SuperMix, 1 mM of each primer, 10 ng of ten-fold diluted DNA template, and 7.0–8.6 L mili-Q water. The following were the PCR thermal cycling conditions: 5 min at 95 °C for initial denaturation; 40 cycles of 5 s at 95 °C and 30 s at 58 °C; and a final step of 40 s at 72 °C. Melting curve analysis was performed after the reactions, with temperatures ranging from 50 to 99 °C. Standard curves were produced, and products were combined in equal parts. The Illumina MiSeq300 platform (Illumina, San Diego, CA, USA) was used for library preparation and sequencing.

#### 2.3.3. Data Sequencing Analysis

The initial findings were analyzed with the Quantitative Insights into Microbial Ecology (QIIME 1.9.0) software [52]. UCLUST grouped high-quality sequences into operational Taxonomic Units (OTUs) at a 97% similarity criterion, in accord with the method described by Edgar [52]. All singleton and chimera OTUs were eliminated. The USEARCH (version 10, https://www.drive5.com/usearch/, accessed on 17 June 2024) program was used to identify and delete chimeric sequences [52]. The RDF classifier was used to perform taxonomic classification on representative sequences from each out, using the method of Cole et al. [53]. Species richness rarefaction curves were plotted against the number of sequences using the Microbiome Analyst [54].

#### 2.3.4. Cd Determination in Plants

The oven-dried samples were crushed into powder and processed with HNO_3_ and HClO_4_ in a 4:1 (*v*/*v*) ratio, generating dilutions of up to 25 mL. Finally, the Cd concentrations in the tissues of plants (roots, shoots, and grains) were evaluated above dilutions using a flame atomic absorption spectrometer (AAA 6300C, Shimadzu, Kyoto, Japan) which has a detection limit of 0.007 µg/mL, in accord with the previously reported procedure [55].

#### 2.3.5. Grain Yield

The rice crop was harvested from every pot, and the grain yields were weighed; mean data were recorded based on three replicated samples. The dry weights of the rice grains were calculated using an appropriate moisture level of 14%.

#### 2.3.6. Bioinformatics and Statistical Analysis

We measured soil microbial diversity indices by using the MOTHUR pipeline (version: v1.40.0), as recommended by Schloss et al. [56]. A Venn diagram was created using the R package “VennDiagram (v. 1.3.3250.34910; http://www.pnl.gov/; http://omics.pnl.gov/, accessed on 17 June 2024)”, in accord with Zaura et al. [57]. A Venn diagram was utilized to demonstrate the amounts of common and unique operational taxonomic units (OTUs) in the samples and to assess the similarity and overlap in the number of OTUs among the samples. Furthermore, redundancy analysis (RDA) was conducted using the software package canoco5 (version 5.0, Microcomputer Power, Ithaca, NY, USA) to investigate the relationship between soil environmental parameters and the functional group of the soil fungal community composition. Using Statistics 8.1 (Tallahassee, FL, USA), an analysis of variance (ANOVA) was performed to determine variations in soil environmental characteristics, Cd levels, and rice grain yield (Table S3). The obtained data were first checked for normalcy. Data in percentages were arcsine-transformed before analysis to normalize the variables. Significant differences (*p* < 0.05) among means were determined using the least significant difference (LSD) with three replicates. All the data are presented as the mean values of three replicates.

## 3. Results

### 3.1. Changes in Soil Chemical Properties and Microbial Biomass

The addition of VC significantly improved the soils’ biochemical traits, such as soil pH, AN, TP, SOC, MBC, and MBN, compared to positive Cd treatment: Pos-Cd + VC1, as shown in Table 2. The VC amendments counteracted the antagonistic effects of Cd on soil quality, and the effect was most significant, for all tested parameters, in Cd toxicity soil with a high VC input. Of the treatments, the higher values were observed in Neg-Cd + VC3 pots. The lower pH, TN, AN, SOC, MBC, and MBN values were noted in sole Pos-Cd pots: Pos-Cd + VC1. Relative to Pos-Cd + VC1, Pos-Cd + VC3 enhanced soil SOC, pH, TN, AN, MBC, and MBN by 5.75%, 41.15%, 18.51%, 12.31%, 60.42%, and 68.37%, correspondingly. Likewise, low VC input also increased each soil quality attribute, but not to the same levels as seen in highly VC-treated pots.

### 3.2. Fungal Community Size and Diversity

**Figure 1:**
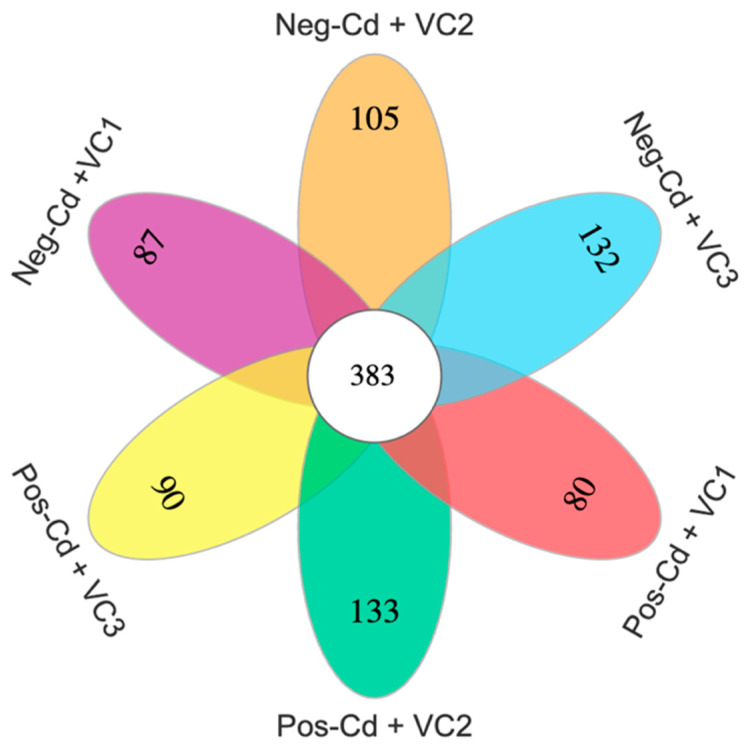
Venn diagram showing the soil fungal unique and shared operational OTUs in a Cd-contaminated soil under vermicompost application. Note: Please see Table 1 for detailed treatment combinations.

**Figure 2:**
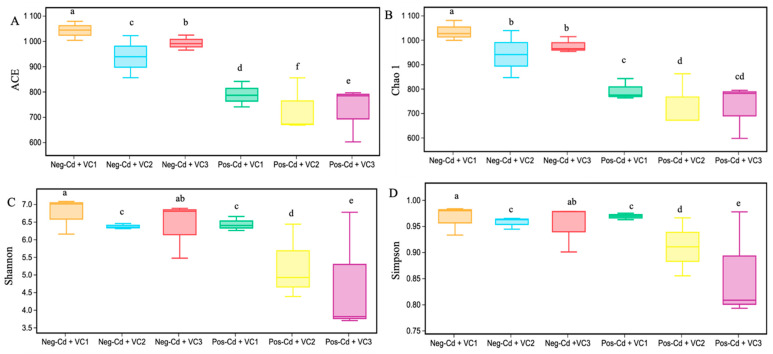
Influence of vermicompost application in a Cd-contaminated soil on fungal *α*-diversity. The different boxes for each treatment represent *α*-diversity estimated by ACE (**A**), Chao 1 (**B**), Shannon (**C**), and Simpson (**D**) indices. The bars represent the standard error of the mean, with different letters indicating a statistical difference at *p* < 0.05. Note: Please see Table 1 for detailed treatment combinations.

In this work, VC amendments considerably improved the soil diversity and structure of the fungal community under Cd stress conditions. The total number of OTUs for Neg-Cd + VC1, Neg-Cd + VC2, Neg-Cd + VC3, Pos-Cd + VC1, Pos-Cd + VC2, and Pos-Cd + VC3 treatments are 940, 824, 884, 716, 665, and 665, respectively (Table S4). The high coverage demonstrated 92% for all six treatments, showing that this sequencing method effectively captured the microbial OTUs in each sample. The Venn diagram shows that the number of unique OTUs in Neg-Cd + VC1, Neg-Cd + VC2, Neg-Cd + VC3, Pos-Cd + VC1, Pos-Cd + VC2, and Pos-Cd + VC3 treatments were 87, 105, 132, 80, 133, and 90, respectively, with 383 common OTUs (Figure 1). The box plot based on the Simpson, Shannon, Chao1, and ACE indices revealed that substantial differences in soil fungal diversity were identified in VC amendments under Cd toxicity (Figure 2).

Compared to Pos-Cd pots, soil treated with VC additions produced greater fungal diversity and abundance. The Neg-Cd treatments had a higher number of community richness indices (ACE and Chao1) than did the Pos-Cd treatments. Furthermore, Neg-Cd treatments produced better community diversity indices (Shannon and Simpson) than Pos-Cd pots.

### 3.3. Microbial Community Composition and Abundance

**Figure 3:**
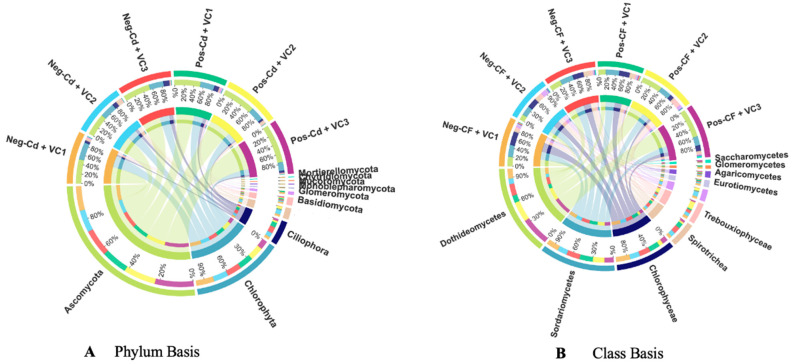
Circos diagram showing the relative abundance of the soil microbial communities at the phylum level (**A**) and class level (**B**), as affected by vermicompost application in Cd-contaminated soils. Note: Please see Table 1 for detailed treatment combinations.

The Circos diagram shows the relative abundance of the fungal community at the phylum and class level, as affected by vermicompost application, in Cd-contaminated soils (Figure 3A,B). The analysis of the microbial community in a Cd-contaminated soil indicated that, on a phylum basis, *Ascomycota*, *Chlorophyta*, *Ciliophora*, *Basidiomycota*, and *Glomeromycta* were the six dominant phyla in soil. *Ascomycota* had the highest abundance in all groups 51% for Neg-Cd + VC1, 42% for Neg-Cd + VC2, 58% for Neg-Cd + VC3, 56% for Pos-Cd + VC1, 60% for Neg-Cd + VC2, and 78% for Pos-Cd + VC3. Similarly, *Dothideomycetes*, *Sordariomycetes*, *Chlorophyceae*, *Spirotrichea*, and *Trebouxiophyceae* were the six dominant microbes on a class basis. *Dothideomycetes* had the greatest abundance in all groups, especially under Cd stress soil: 20% for Neg-Cd + VC1, 13% for Neg-Cd + VC2, 22% for Neg-Cd + VC3, 21% for Pos-Cd + VC1, 40% for Neg-Cd + VC2, and 60% for Pos-Cd + VC3. The outcomes exhibited that the application of VC improved the composition and abundance of the soil fungal community on the phylum and class levels in a Cd-contaminated soil.

### 3.4. Relative Influence of Soil Properties and Cd Toxicity on Fungal Community

**Figure 4:**
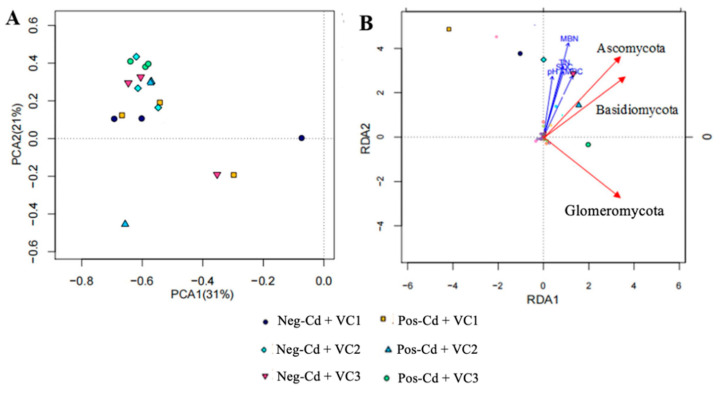
Principal component analyses (PCA) of soil fungal communities: (**A**) soil sampled from each sample, and results from the RDA (**B**), seeking to explore the relationship among fungal communities and soil biochemical traits. Note: Please see Table 1 for detailed treatment combinations.

We used principal coordinate analyses (PCA) based on the weighted Fast UniFrac metrics to investigate the variance in fungal communities, as impacted by VC application under Cd toxicity (Figure 4A). The first-principle coordination axis accounted for 31%, whereas the second key coordinating axis in fungal communities contributed 21%. The PCA revealed substantial differences in fungal communities between VC and Cd additions: Neg-Cd + VC3, Neg-Cd + VC2, Pos-Cd + VC1, and Pos-Cd + VC2 on the positive side of the x-axis; and Neg-Cd + VC3, Pos-Cd + VC1, and Pos-Cd + VC2 on the negative side.

The RDA was conducted, based on phylum, between different treatments to explore the relative levels of influence of soil biochemical attributes and Cd toxicity on the abundance and diversity of the fungal community (Figure 4B). The RDA revealed that the soil’s chemical traits and microbial biomass N and C strongly contributed to fungal communities. The analysis explains the six treatments placed in different sections, reporting that the VC had a significant effect on the structure of fungal communities and soil biochemical attributes (SOC, pH, AN, TN, MBN and MBC). In addition, the RDA further exhibited that VC amendments significantly changed fungal communities to a greater degree than seen in negative VC pots, in Cd-contaminated soil. These results show that VC application to paddy fields significantly impacts soil fungal community richness, composition, and nutrient levels. The finding exhibited the fact that the soil’s traits played a vital role in structuring the soil’s fungal communities.

Moreover, for more detailed information, correlation analyses among soil biochemical attributes and fungal phyla were conducted using Pearson correlation analysis (Figure S2). The soil’s environmental traits were highly positively associated with dominant fungal phyla. The abundance of dominant phyla *Ascomycota* and *Basidiomycota* were strongly allied with soil microbial biomass N and C and chemical properties. The analyses further showed that the soil biochemical traits significantly affected the fungal community abundance and competition. Moreover, heatmap analysis explored the relationship between different VC + Cd amendments and soil fungal communities (Figure S3). The relationships of different VC fertilization treatments, especially high-VC treatments, such as Pos-Cd + VC3, with the top fungal phyla were highly positively correlated under Cd toxicity. As a result, various VC amendments modified the fungal community diversity and compositional pattern by affecting the soil’s physical and biochemical traits under Cd stress conditions.

### 3.5. Effect of VC on Cd Accumulation and Rice Yields

Cd poisoning significantly enhanced Cd accumulation in aromatic-rice plant sections, resulting in lower rice grain production (Table 3). However, using vermicompost reduced Cd-induced toxicity, significantly increased rice grain production, and lowered Cd uptake and accumulation. The high-VC: Neg-Cd + VC3 supply produced the highest values among the treatments. while the Pos-Cd + VC1 treatment had the lowest grain yield. In contrast to Pos-Cd + VC1, the high-VC treatment (Pos-Cd + VC3) improved rice grain production by 37.76% under Cd toxicity. Similarly, low-VC-supply treatments raised each tested variable, albeit not as consistently as high-VC treatments. Furthermore, rice plants absorbed and accumulated more Cd in Cd-contaminated soil, showing the order: root > stem + leaves > grains. The use of VC considerably lowered Cd concentrations in rice roots, leaves + stem, and grain. The results demonstrated that a high-VC (6 tone ha^−1^) treatment significantly reduced Cd accumulation and concentrations in rice plants under Cd toxicity. Thus, the use of VC mitigated the harmful effects of Cd stress on rice yield and production.

## 4. Discussion

Heavy-metal pollution, especially of Cd, is always found around smelting, mining, waste recycling, and manufacturing sites [3,4]. Continued phosphate fertilizer application has caused serious Cd contamination in arable land [7,13]. The biochemical properties of soil, fungal community structure, and diversity are essential signs of soil biology, as they enhance soil quality and functionality and rice yield [2,18]. It is well documented that soil microbes are sensitive to pollution, especially Cd toxicity [13,14]. In this work, we assumed that the VC application counteracted the negative effects of Cd toxicity on paddy soil biochemical traits, fungal community structure and diversity, and grain yield.

### 4.1. Soil Biochemical Attributes

In this work, VC supply enhanced soil chemical attributes, i.e., SOC, AN, TN, pH, and microbial biomass N and C in Cd-contaminated soils (Table 2). We determined that enhancing the dose of VC increases soil quality and progressively releases essential plant nutrients in the soil. Applying VC improved the pH levels of soils considerably, compared to Neg-Cd pots. According to Ni et al. [58], a decrease in soil pH is triggered by the excessive use of chemical nitrogen, which produces H+ via nitrification. Another element that could contribute to soil acidity induced by chemical fertilizer application involved the acidic qualities of chemical nitrogen fertilizer, which could lead to a lower pH value for the soil [59]. However, using organic fertilizers considerably increased the soil pH, as stated in the earlier studies [17,35]. Similarly, in our investigation, vermicompost significantly raised soil pH (Table 2). Hydroxyl ions (OH-) from the negatively charged functional group in vermicompost and breakdown of CaCO_3_ or CaO might lead to higher soil pH by interacting with H+ ions [60].

Additionally, VC application significantly increased soil organic C, TN, and AN content, compared to negative VC treatment (Table 2). VC is high in organic matter, and the plant demands nutrients, which could explain why SOC, TN, and AN levels increased in this study’s high-VC treatments. Similar results have been reported, indicating that the use of VC greatly improved soil health due to its nutrient wealth [19]. According to Liang et al. [61], heavy metals do not dissolve or migrate easily in high-pH soils. Thus, in this study, higher soil pH following VC fertilizer treatment may have played an important role in slowing Cd migration in soil. Likewise, improved soil nutrients promote healthy plant growth. In summary, VC may improve soil health and fertility while reducing the movement of Cd and other heavy metals.

The current investigation found that a high dose of VC boosted microbial biomass N and C under Cd toxicity (Table 2). Improvements in soil microbial biomass might have occurred due to the VC’s having improved the soil’s biogeochemical characteristics, resulting in improved plant absorption of inorganic N [17,61]. Another reason could be that VC application increased soil nutrient content and crop biomass collection, hence increasing crop residues [62]. Furthermore, VC additions significantly raised soil MBN and MBC in this work, compared to non-VC-treated soil. This disparity could be explained by the fact that the VC included a considerable amount of carbon, which could fuel microbe activity and growth [63].

### 4.2. Soil Fungal Diversity and Composition

Microbes are important indicators of soil quality and fertility [64]. Conventional farming methods typically lower the value and diversity of the fungal population size, a level which is critical for soil activity, reliability, and long-term survival [2,17]. In this study, the application of VC had a significant impact on the variety and abundance of the fungal community under Cd toxicity. Weifeng et al. [65] reported that organic additions include more substrates for microbial activity than found in synthetic fertilizer applications. They also bring microorganisms present in organic additions directly into the soil. Microbial abundance was strongly linked with SOC concentration (Figure S3), indicating that the application of organic amendments enhanced fungal substrates, promoting fungal population, which may, in turn, enhance fungal activity and function in nutrient cycling [66]. This study also discovered a strong correlation between soil fungus diversity and soil pH (Figure S2). Acidic soils may increase ecosystem filtering through habitat selection, disrupting the balance of fungal taxa in their native habitat and resulting in the extinction of some taxa [67]. A recent study found that soil pH is the primary determinant of microbial diversity and structure, with a neutral pH showing the greatest richness and diversity of microbes [68].

Our findings revealed that the soil fungal community altered considerably under Cd toxicity (Figure 3). Previous reports also have established the adverse influences of heavy metals on the assembling of soil fungal communities [67,69]. The presence of heavy metals can have a deleterious impact on the development, survival, and composition of soil microbial communities [14]. According to Song et al. [16], Cd stress reduced the microbial community composition in underlying soils. In this regard, VC, which stabilizes organic materials through the interactions of microbes and earthworms, has been considered a helpful asset for soil restoration and improving C content and soil quality [25]. Several research efforts have revealed that the application of VC in agricultural land has resulted in increased soil productivity and yield and increased microbial functional diversity [25,70]. The soil fungal community structure and composition were considerably (*p* < 0.05) enhanced by VC amendments under Cd toxicity conditions; VC application counteracted the adverse effects of Cd on the fungal community in the current study (Figure 3). Earlier studies also determined the differing reactions of the fungal community to organic amendment applications [71,72]. An analysis of soil microbial community under VC application in a Cd-contaminated soil indicated that *Ascomycota*, *Chlorophyta*, *Ciliophora*, *Basidiomycota*, and *Glomeromycta* were the dominant microbial phyla in soil on a phylum basis. The application of VC significantly increased the dominant fungal phyla in Cd-contaminated soil, compared to harmful VC soil (Figure 3).

Correlation analyses showed highly positive relationships among the different VC amendments with soil environmental factors and fungal communities. The PCA showed that the different VC amendments significantly altered the fungal community structure (Figure 4A). In addition, the RDA also revealed that the different VC amendments positively correlated with soil environmental factors and dominant fungal phyla (Figure 4B). Wang et al. [19] stated that the VC application can alter soil’s biochemical attributes, changing microbial community structure and diversity. Similarly, in another article, Iqbal et al. [35] stated that the microbial community is highly positively correlated to soil’s biochemical traits. The leading fungal phyla, particularly *Ascomycota*, *Basidiomycota*, and *Glomeromycta*, were positively correlated with soil environmental variables. Previous research found that soil pH was not only highly related to microbial communities and diversity [35], but it also had a positive relationship with soil fungal abundance; therefore, pH was an essential variable in defining the abundance and distribution of soil fungal community.

Furthermore, the abundance of the fungal community was found to have strong connections with microbial biomass C and N and soil nutrients (Table 2). These data suggested that nutritional element levels were the critical edaphic criteria influencing the makeup of fungal communities. Because soil C and N are microorganisms’ primary energy bases and component materials, they affect soil microbes’ spread by maintaining their metabolism [73]. According to the findings stated above, using vermicompost may create a favorite growth habitat for soil microorganisms, improving microbial community composition and health.

### 4.3. Rice Yield and Cd Uptake and Accumulation

The VC significantly improved rice grain yield and decreased the Cd uptake in rice organs compared to the control (Table 2). Visible increases in rice yield are highly associated with improvements in soil physiochemical and biological characteristics under organic amendment application [66,74]. Organic fertilizers improve soil quality and health, hence increasing plant growth, crop production, and yield factors [17,75]. In the present investigation, vermicompost-treated pots had higher soil microbial biomass C and N, as well as increases in nutritional contents (Table 2), which aided aromatic-rice growth and production by providing sufficient nutrients across the growing season.

Additionally, organic additions reduced the concentration of Cd in fragrant-rice tissues while considerably increasing rice yields, demonstrating that the amendments used in our study were still effective in decreasing Cd uptake and accumulation (Table 3). Changes in plant biomass, Cd Phyto availability, and soil environmental variables may have contributed to this decrease. Our findings showed that after VC amendments, a decline in rice grain Cd content was accompanied by increased grain yields, resulting in the so-called bio-dilution effect, a finding consistent with an earlier study [76]. Furthermore, organic amendments aided in retaining or immobilizing significant levels of Cd in the root, which has been thought to be a critical procedure for shielding plants against Cd dissemination [77]. Once the plant root absorbs Cd, it may be transported to the developing grain via one of two routes: (1) Cd is transmitted by uptake and directly transported to the building grain via the xylem, or (2) Cd is transported through actively transpiring parts (such as rachis, leaves, culms, and panicles), and then rapidly remobilized to grains via the phloem [77]. We found in the current investigation that the VC additions effectively reduced Cd uptake and translocation from roots and stems to grains, implying that the organic amendments utilized in this work could minimize Cd uptake by grain.

## 5. Conclusions

In this study, the application of VC significantly improved the soil fertility, fungal community diversity and richness, and grain yield of fragrant rice under Cd stress conditions. Moreover, VC supply decreased the Cd uptake and the accumulation of Cd in fragrant rice’s different organs, including roots, shoots, and grains. The main reasons for the reduced Cd uptake in rice are that VC amendments influence the soil’s biological traits, reducing Cd availability, and this subsequently impacts Cd uptake and transportation in the different organs of rice. Cd availability is reduced in soil, resulting in the decreased uptake and translocation of Cd from the roots to the shoots and grain. In conclusion, our findings show that the use of VC significantly improves soil health and fragrant-rice production under Cd toxicity.

## Figures and Tables

**Table 1:** The detailed treatment combinations used in this experiment.

No.	Treatment Name	Treatment Combination
1	Neg-Cd + VC1	0 mg Cd + 0 t ha^−1^ VC
2	Neg-Cd + VC2	0 mg Cd + 3 t ha^−1^ VC
3	Neg-Cd + VC3	0 mg Cd + 6 t ha^−1^ VC
4	Pos-Cd + VC1	25 mg Cd + 0 t ha^−1^ VC
5	Pos-Cd + VC2	25 mg Cd + 3 t ha^−1^ VC
6	Pos-Cd + VC3	25 mg Cd + 6 t ha^−1^ VC

Note: Neg, negative; Pos, positive; Cd, cadmium; VC, vermicompost.

**Table 2:** Influence of vermicompost on soil microbial biomass and chemical traits under Cd toxicity.

Treatment	pH	SOC (g kg^−1^)	TN (g kg^−1^)	AN (mg kg^−1^)	MBC (mg kg^−1^)	MBN (mg kg^−1^)
Neg-Cd+ VC1	5.95 ± 0.86 e	11.25 ± 1.46 e	1.12 ± 0.16 d	142.50 ± 13.50 d	214.45 ± 12.34 c	41.45 ± 2.15 c
Neg-Cd +VC2	6.25 ± 0.56 b	14.34 ± 1.22 c	1.25 ± 0.82 b	154.09 ±15.63 b	270.33 ± 14.34 b	55.56 ± 2.55 b
Neg-Cd +VC3	6.25 ± 0.66 a	16.44 ± 2.12 a	1.36 ± 0.10 a	178.52 ± 13.76 a	321.35 ± 18.34 a	60.32 ± 4.21 a
Pos-Cd + VC1	5.92 ± 0.70 d	10.22 ± 1.96 d	1.07 ± 0.06 e	136.15 ± 10.70 e	130.23 ± 10.23 e	25.34 ± 3.07 d
Pos-Cd +VC2	6.02 ± 0.35 c	12.26 ± 1.76 d	1.12 ± 0.07 c	150.51 ± 21.52 c	190.56 ± 9.48 d	39.45 ± 5.62 d
Pos-Cd + VC3	6.24 ± 0.54 b	14.45 ± 2.02 b	1.29 ± 0.16 b	156.02 ± 17.50 b	208.65 ± 15.33 c	42.60 ± 3.82 c

Note: Cd, cadmium; VC, vermicompost; SOC, soil organic carbon; TN, total nitrogen, AN, available nitrogen; MBC, microbial biomass carbon; MBN, microbial biomass nitrogen. The least significant difference (LSD) test was used to evaluate treatment means and for different lettering, and lettering was performed at 5%. The statistics show that values in columns with the same letters are statistically the same at (*p* < 0.05). Please see Table 1 for detailed treatment combinations.

**Table 3:** Effects of vermicompost application on Cd accumulation in different parts of rice plants, and grain yield under Cd toxicity.

	Cd Content (µg g^−1^ DW)			
Treatments	Root	Stem + Leaf	Grain	Grain Yield (kg ha^−1^)
Neg-Cd + VC1	35.98 ± 4.46 c	12.98 ± 1.12 d	0.15 ± 0.01 d	77.08 ± 4.45 d
Neg-Cd + VC2	26.87 ± 2.32 d	7.87 ± 0.87 e	0.11 ± 0.01 d	98.86 ± 8.88 b
Neg-Cd + VC3	12.75 ± 1.68 e	3.46 ± 0.44 f	0.12 ± 0.02 e	121.36 ± 15.45 a
Pos-Cd + VC1	189.75 ± 12.44 a	46.87 ± 3.44 a	1.44 ± 0.07 a	62.45 ± 5.76 e
Pos-Cd + VC2	135.98 ± 9.34 b	23.87 ± 2.32 b	0.87 ± 0.04 b	71.44 ± 9.65 c
Pos-Cd + VC3	116.45 ± 12.30 b	16.98 ± 1.88 c	0.45 ± 0.03 c	95.36 ± 8.25 b

Note: Cd, Cadmium; VC, vermicompost. The least significant difference (LSD) test was utilized for distinct groups, and lettering was performed at 5%. Statistics show that values in a column with the same letters are statistically equivalent at (*p* < 0.05). Note: Please see Table 1 for detailed treatment combinations.

## Data Availability

The original contributions presented in the study are included in the article/supplementary material, further inquiries can be directed to the corresponding author.

## Supplementary Materials

The following supporting information can be downloaded at: https://www.mdpi.com/article/10.3390/microorganisms12061252/s1, Figure S1: Experimental rice plant pots pictures including different treatment pots; Figure S2: Correlation analyses between soil environmental variables and top phyla at the phylum level. The right side of the legend shows the color range and the corresponding correlation value. Note: * = Significance level at 0.05, and ** = Significance level at 0.001; Figure S3: Heatmap correlation analysis between different treatments and soil and top fungal phyla at the phylum level. The right side of the legend shows the color range and the corresponding correlation value; Table S1: Soil physiochemical attributes before experimentation; Table S2: The meteorological data during the experimental time; Table S3: Analysis of variance (ANOVA) for soil and rice physio-biochemical traits and grain yield; Table S4: The total number of operational taxonomic units in replicated form.
