# Supplementary material for: Vermicompost Supply Enhances Fragrant-Rice Yield by Improving Soil Fertility and Eukaryotic Microbial Community Composition under Environmental Stress Conditions
Source: Microorganisms. 2024 Jun 20;12(6):1252. doi: 10.3390/microorganisms12061252 (PMC11206116; doi:10.3390/microorganisms12061252)
Supplement: Supplementary file 1 [file microorganisms-12-01252-s001.zip › microorganisms-3021332-supplementary.pdf]

Supplementary Data

Table S1. Soil and vermicompost physiochemical attributes before experimentation

Soil Properties	Content
Soil organic carbon (g kg^{-1})	9.05
Total nitrogen (g kg^{-1})	0.95
Total phosphorus (g kg^{-1})	0.88
Total potassium (g kg^{-1})	4.22
pH	5.96
Available nitrogen (mg kg^{-1})	144.5
Available phosphorus (mg kg^{-1})	15.3
Available potassium (mg kg^{-1})	125.2
Cadmium (mg kg^{-1})	2.1

Table S2. The meteorological data during the experimental time.

**Month**	**Average temperature (℃)**	**Precipitation (mm)**	**Humidity (%)**
July	28.50	325.55	84.45
August	28.65	198.45	82.65
September	28.20	242.85	83.25
October	24.50	28.88	70.16
November	19.60	62.65	75.2

Table S3. Analysis of variance (ANOVA) for soil and rice physio-biochemical traits and grain yield.

Parameters	df	SS	MS	F	P
pH	5	0.36	0.07	651**	0.00
SOC	5	131.75	26.93	4.5*	0.02
TN	5	0.19	0.04	27.9**	0.00
AN	5	3459.32	691.86	16**	0.00
MBC	5	64653.80	12930.80	493**	0.00
MBN	5	2197.06	439.41	64.1**	0.00
Root-Cd	5	76253.50	15250.70	905**	0.00
Shoot-Cd	5	3298.18	659.64	493**	0.00
Grain-Cd	5	4.28	0.86	3350**	0.00
GY	5	7313.64	1462.73	78.8**	0.00

Note: SOC, soil organic carbon; TN, total nitrogen; AN, available nitrogen; MBC, microbial biomass carbon; MBN, microbial biomass nitrogen; Cd, cadmium; GY grain yield; df, degree of freedom; SS, sum of square; MS, mean of square; * & **Significance level at 0.05 and 0.001, respectively.

Table S4. The total number of operational taxonomic units in replicated form.

SampleID	Total Tags	Taxon Tags	Unclassified Tags	Singleton Tags	OTUs
C1V1-1	127055	119492	5983	1580	955
C1V1-2	108325	104229	2522	1574	958
C1V1-3	122379	118546	2701	1132	907
C1V2-1	121813	116059	4593	1161	903
C1V2-2	114197	98637	14374	1186	728
C1V2-3	117516	111771	4196	1549	842
C1V3-1	121992	118047	1818	2127	858
C1V3-2	106229	101608	2756	1865	910
C1V3-3	120060	111889	5773	2398	884
C2V1-1	113284	106135	6338	811	716
C2V1-2	112921	109701	2361	859	661
C2V1-3	110318	105594	2951	1773	771
C2V2-1	121610	116822	3496	1292	791
C2V2-2	120265	116592	1275	2398	631
C2V2-3	110040	106831	873	2336	636
C2V3-1	127921	126069	245	1607	691
C2V3-2	109884	108236	177	1471	699
C2V3-3	114115	105887	6041	2187	577

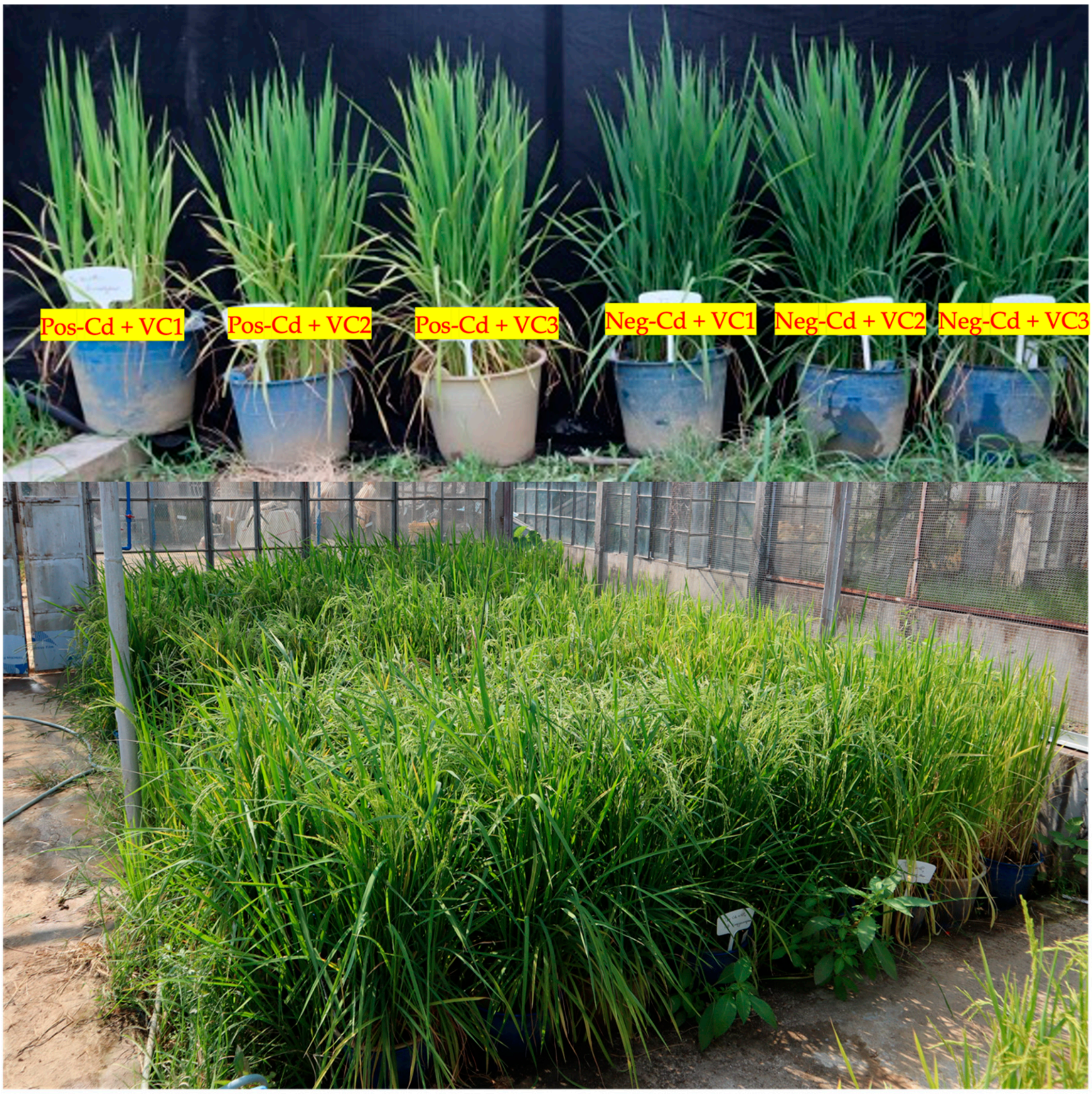

Figure S1. Experimental rice plants pots pictures including treatment names.

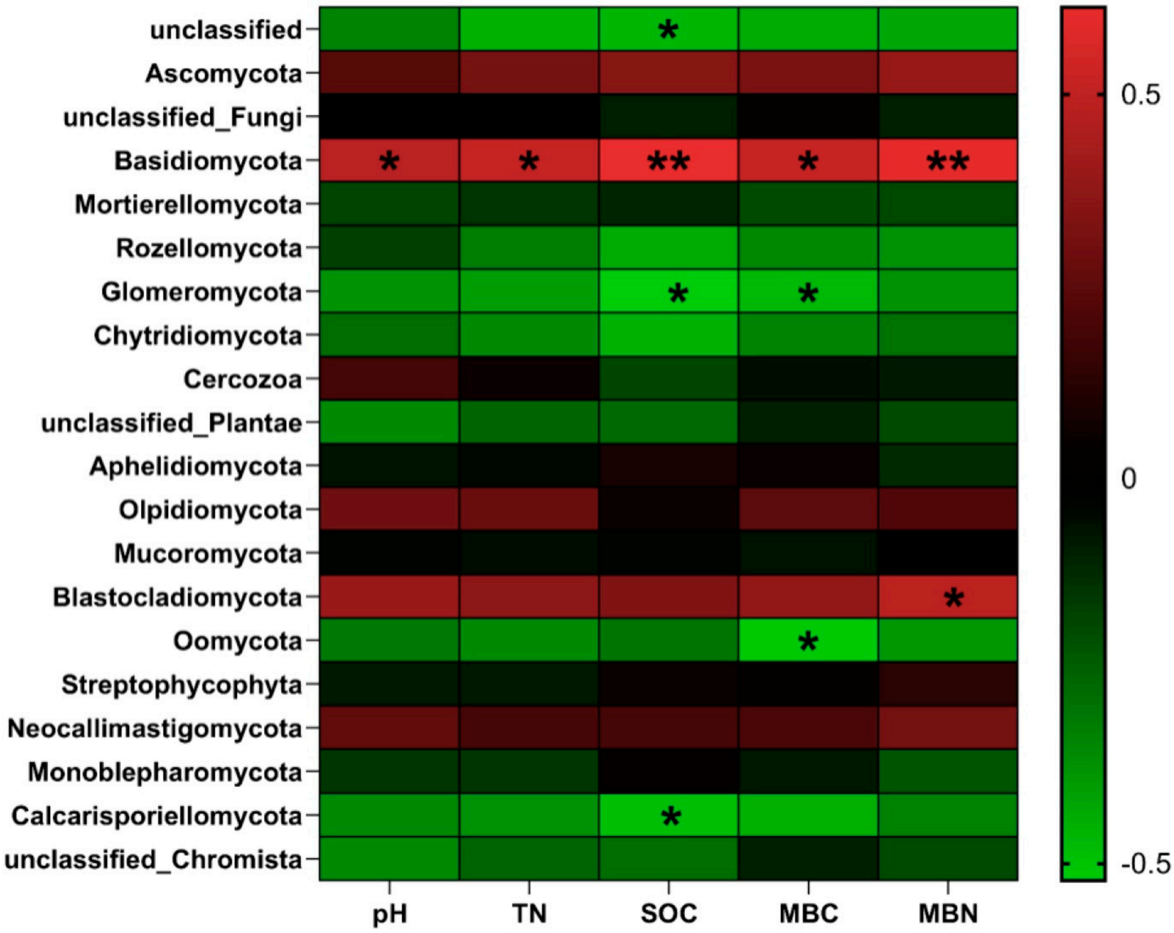

Figure S2. Correlation analyses between soil environmental variables and top phyla at the phylum level. The right side of the legend shows the color range and the corresponding correlation value. Note: *= Significance level at 0.05, and **=Significance level at 0.001.

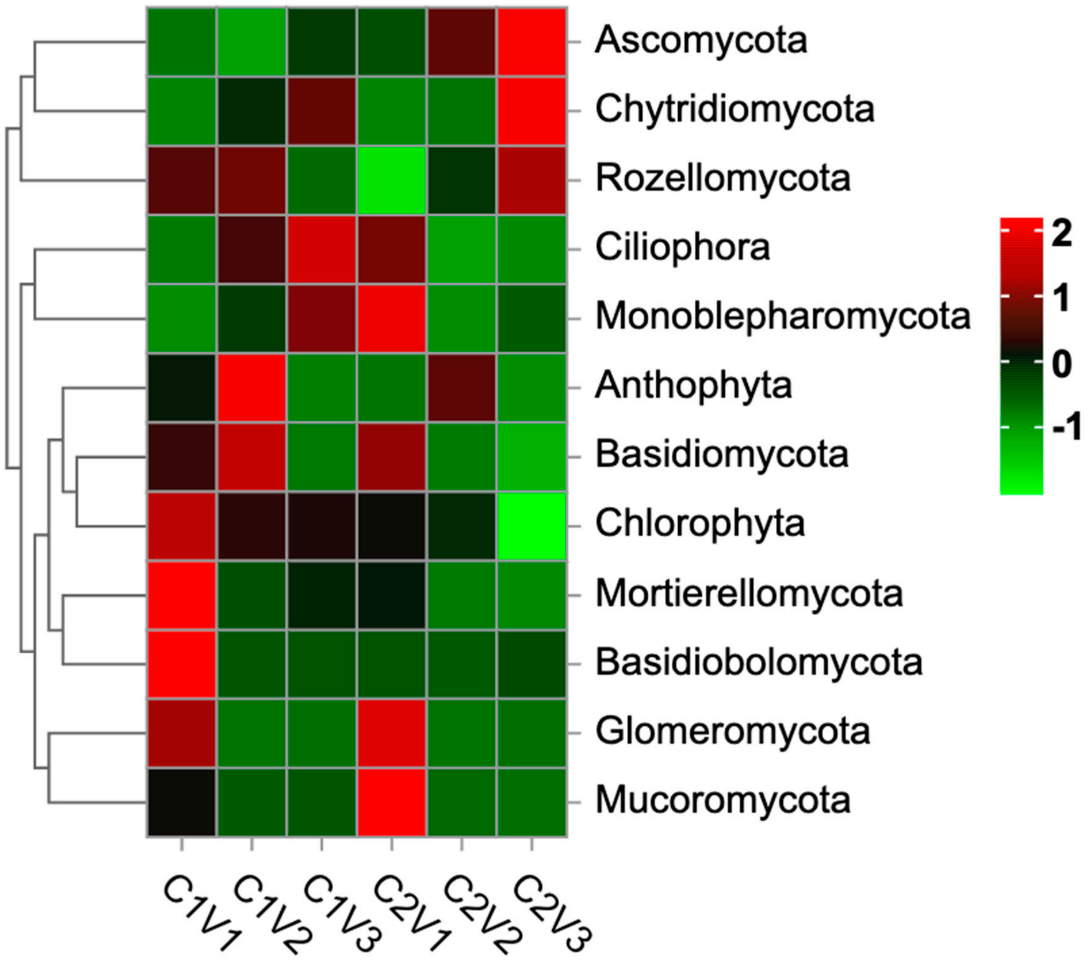

Figure S3. Heatmap correlation analysis between different treatments and soil and top fungal phyla at the phylum level. The right side of the legend shows the color range and the corresponding correlation value.
